# Supplementary material for: Deep-ocean macrofaunal assemblages on ferromanganese and phosphorite-rich substrates in the Southern California Borderland
Source: PeerJ. 2024 Oct 31;12:e18290. doi: 10.7717/peerj.18290 (PMC11531752; doi:10.7717/peerj.18290)
Supplement: Supplemental Information 1 [file peerj-12-18290-s001.docx]

Table A1. Macrofaunal abundance per rock, macrofaunal density per 200 cm^2^, and surface area of each rock collected. Sample number, dive number, rock number and site are provided for context.

| Sample | Dive | Rock | Site | Macrofauna count | Macrofauna density (ind. 200 cm-2) | Surface Area (cm^2^) | Substrate type |
| --- | --- | --- | --- | --- | --- | --- | --- |
| HD1840-R2 | HD1840 | 2 | Patton Escarpment Central | 48 | 14.28 | 671.97 | Basalt |
| HD1840-R3 | HD1840 | 3 | Patton Escarpment Central | 73 | 9.95 | 1466.76 | Phosphorite |
| HD1840-R4 | HD1840 | 4 | Patton Escarpment Central | 20 | 11.20 | 356.94 | Sedimentary |
| HD1840-R5 | HD1840 | 5 | Patton Escarpment Central | 42 | 10.56 | 794.80 | Sedimentary |
| HD1840-R6 | HD1840 | 6 | Patton Escarpment Central | 41 | 11.69 | 700.87 | Sedimentary |
| HD1841-R1 | HD1841 | 1 | San Juan Seamount Upper Flank | 13 | 3.34 | 777.46 | FeMn crust |
| HD1841-R3 | HD1841 | 3 | San Juan Seamount Upper Flank | 22 | 8.22 | 534.68 | FeMn crust |
| HD1841-R4 | HD1841 | 4 | San Juan Seamount Upper Flank | 35 | 7.55 | 926.30 | FeMn crust |
| HD1841-R5 | HD1841 | 5 | San Juan Seamount Upper Flank | 85 | 14.03 | 1210.98 | FeMn crust |
| HD1841-R6 | HD1841 | 6 | San Juan Seamount Upper Flank | 70 | 10.62 | 1317.92 | FeMn crust |
| HD1842-R1 | HD1842 | 1 | Northeast Bank | 236 | 29.72 | 1588.15 | FeMn crust |
| HD1842-R2 | HD1842 | 2 | Northeast Bank | 15 | 7.46 | 401.73 | FeMn crust |
| HD1842-R3 | HD1842 | 3 | Northeast Bank | 27 | 6.25 | 862.72 | Basalt |
| HD1842-R4 | HD1842 | 4 | Northeast Bank | 20 | 7.07 | 565.03 | FeMn crust |
| HD1842-R5 | HD1842 | 5 | Northeast Bank | 20 | 7.22 | 553.47 | Basalt |
| HD1843-R1 | HD1843 | 1 | Cortes Bank | 141 | 19.61 | 1437.86 | Phosphorite |
| HD1843-R3 | HD1843 | 3 | Cortes Bank | 196 | 19.96 | 1963.87 | Phosphorite |
| HD1843-R4 | HD1843 | 4 | Cortes Bank | 46 | 24.20 | 380.06 | Phosphorite |
| HD1843-R5 | HD1843 | 5 | Cortes Bank | 41 | 19.36 | 423.41 | Phosphorite |
| HD1843-R6 | HD1843 | 6 | Cortes Bank | 40 | 19.15 | 417.63 | Phosphorite |
| HD1844-R2 | HD1844 | 2 | Patton Ridge South | 33 | 9.82 | 671.97 | Phosphorite |
| HD1844-R3 | HD1844 | 3 | Patton Ridge South | 61 | 12.94 | 942.20 | Phosphorite |
| HD1844-R4 | HD1844 | 4 | Patton Ridge South | 39 | 10.18 | 765.90 | Phosphorite |
| HD1844-R6 | HD1844 | 6 | Patton Ridge South | 51 | 9.26 | 1101.16 | Phosphorite |
| HD1844-R7 | HD1844 | 7 | Patton Ridge South | 29 | 5.77 | 1004.34 | Phosphorite |
| HD1845-R1 | HD1845 | 1 | 40-Mile Bank | 19 | 10.91 | 348.27 | Sedimentary |
| HD1845-R2 | HD1845 | 2 | 40-Mile Bank | 18 | 2.71 | 1328.03 | FeMn crust |
| HD1845-R3 | HD1845 | 3 | 40-Mile Bank | 10 | 3.74 | 534.68 | FeMn crust |
| HD1845-R5 | HD1845 | 5 | 40-Mile Bank | 34 | 7.82 | 868.50 | Sedimentary |
| HD1845-R6 | HD1845 | 6 | 40-Mile Bank | 47 | 7.45 | 1260.12 | Sedimentary |
| HD1846-R1 | HD1846 | 1 | San Clemente Escarpment | 14 | 4.32 | 647.40 | FeMn crust |
| HD1846-R2 | HD1846 | 2 | San Clemente Escarpment | 39 | 9.19 | 848.27 | Basalt |
| HD1846-R3 | HD1846 | 3 | San Clemente Escarpment | 33 | 8.50 | 776.01 | FeMn crust |
| HD1846-R4 | HD1846 | 4 | San Clemente Escarpment | 12 | 3.45 | 695.09 | FeMn crust |
| HD1846-R6 | HD1846 | 6 | San Clemente Escarpment | 12 | 5.22 | 459.54 | FeMn crust |
| HD1847-R2 | HD1847 | 2 | Osborn Bank Meso | 58 | 15.55 | 745.66 | Basalt |
| HD1847-R3 | HD1847 | 3 | Osborn Bank Meso | 34 | 15.58 | 436.42 | Basalt |
| HD1847-R5 | HD1847 | 5 | Osborn Bank Meso | 63 | 14.50 | 868.50 | Basalt |
| HD1847-R7 | HD1847 | 7 | Osborn Bank Meso | 45 | 20.15 | 446.53 | Basalt |
| SCB-006 | S0440 | 1 | Hancock Bank | 39 | 14.81 | 526.56 | Basalt |
| SCB-007 | S0440 | 2 | Hancock Bank | 60 | 15.48 | 775 | Phosphorite |
| SCB-010 | S0440 | 3 | Hancock Bank | 45 | 11.73 | 767.18 | Basalt |
| SCB-011 | S0440 | 4 | Hancock Bank | 51 | 26.42 | 385.93 | Basalt |
| SCB-015 | S0440 | 5 | Hancock Bank | 46 | 17.57 | 523.43 | FeMn crust |
| SCB-016 | S0440 | 6 | Hancock Bank | 32 | 23.40 | 273.43 | Basalt |
| SCB-019 | S0440 | 7 | Hancock Bank | 61 | 11.90 | 1025 | Basalt |
| SCB-026 | S0440 | 8 | Hancock Bank | 49 | 10.01 | 978.12 | Basalt |
| SCB-075 | S0443 | 1 | San Juan Seamount North | 6 | 3.02 | 396.87 | FeMn crust |
| SCB-076 | S0443 | 2 | San Juan Seamount North | 24 | 6.88 | 696.87 | FeMn crust |
| SCB-077 | S0443 | 3 | San Juan Seamount North | 8 | 1.69 | 945.31 | FeMn crust |
| SCB-085 | S0443 | 4 | San Juan Seamount North | 29 | 7.42 | 781.25 | FeMn crust |
| SCB-087 | S0443 | 5 | San Juan Seamount North | 38 | 12.25 | 620.31 | FeMn crust |
| SCB-090 | S0443 | 6 | San Juan Seamount North | 35 | 4.65 | 1504.68 | FeMn crust |
| SCB-095 | S0443 | 7 | San Juan Seamount North | 138 | 31.71 | 870.31 | FeMn crust |
| SCB-096 | S0443 | 8 | San Juan Seamount North | 70 | 24.48 | 571.87 | FeMn crust |
| SCB-099 | S0444 | 1 | Patton Escarpment | 3 | 0.73 | 810.93 | FeMn crust |
| SCB-107 | S0444 | 3 | Patton Escarpment | 2 | 0.72 | 554.68 | FeMn crust |
| SCB-116 | S0444 | 4 | Patton Escarpment | 3 | 1.32 | 451.56 | FeMn crust |
| SCB-117 | S0444 | 5 | Patton Escarpment | 6 | 3.57 | 335.93 | FeMn crust |
| SCB-119 | S0444 | 6 | Patton Escarpment | 19 | 7.48 | 507.81 | FeMn crust |
| SCB-127 | S0444 | 8 | Patton Escarpment | 9 | 4.02 | 446.87 | FeMn crust |
| SCB-134 | S0445 | 1 | Little Joe Seamount | 10 | 4.07 | 490.62 | FeMn crust |
| SCB-135 | S0445 | 2 | Little Joe Seamount | 4 | 0.56 | 1410.93 | FeMn crust |
| SCB-145 | S0445 | 3 | Little Joe Seamount | 4 | 0.76 | 1046.87 | FeMn crust |
| SCB-146 | S0445 | 4 | Little Joe Seamount | 10 | 2.23 | 895.31 | FeMn crust |
| SCB-151 | S0445 | 5 | Little Joe Seamount | 5 | 2.48 | 403.12 | FeMn crust |
| SCB-152 | S0445 | 6 | Little Joe Seamount | 22 | 7.56 | 581.25 | FeMn crust |
| SCB-154 | S0445 | 7 | Little Joe Seamount | 5 | 1.10 | 903.12 | FeMn crust |
| SCB-155 | S0445 | 8 | Little Joe Seamount | 5 | 1.54 | 648.43 | FeMn crust |
| SCB-208 | S0448 | 1 | Crespi Knoll | 229 | 39.77 | 1151.56 | Basalt |
| SCB-218 | S0448 | 3 | Crespi Knoll | 27 | 8.83 | 610.93 | Sedimentary |
| SCB-223 | S0448 | 4 | Crespi Knoll | 38 | 18.92 | 401.56 | Basalt |
| SCB-224 | S0448 | 5 | Crespi Knoll | 39 | 9.65 | 807.81 | Basalt |
| SCB-227 | S0448 | 6 | Crespi Knoll | 31 | 19.16 | 323.43 | Basalt |
| SCB-229 | S0448 | 7 | Crespi Knoll | 15 | 8.27 | 362.50 | Basalt |
| SCB-329 | S0452 | 1 | Coronado Escarpment | 66 | 10.14 | 1301.56 | Phosphorite |
| SCB-330 | S0452 | 2 | Coronado Escarpment | 61 | 11.49 | 1060.93 | Phosphorite |
| SCB-331 | S0452 | 3 | Coronado Escarpment | 79 | 13.77 | 1146.87 | Phosphorite |
| SCB-332 | S0452 | 4 | Coronado Escarpment | 66 | 22.34 | 590.62 | Phosphorite |
| SCB-333 | S0452 | 5 | Coronado Escarpment | 51 | 10.32 | 987.50 | Phosphorite |
| SCB-342 | S0452 | 6 | Coronado Escarpment | 78 | 23.94 | 651.56 | Phosphorite |
| SCB-343 | S0452 | 7 | Coronado Escarpment | 55 | 11.99 | 917.18 | Phosphorite |

Table A2. Diversity metrics calculated per rock.

| Sample | Dive | Rock | Species richness | H'_(loge)_ | H'_(log10)_ | J' | ES_(5)_ | ES_(10)_ | ES_(20)_ |
| --- | --- | --- | --- | --- | --- | --- | --- | --- | --- |
| HD1840-R2 | HD1840 | 2 | 16 | 2.65 | 1.15 | 0.96 | 4.67 | 8.72 | 16.00 |
| HD1840-R3 | HD1840 | 3 | 21 | 2.59 | 1.12 | 0.85 | 4.14 | 7.04 | 11.36 |
| HD1840-R4 | HD1840 | 4 | 8 | 2.03 | 0.88 | 0.97 | 4.56 | 8.00 | 8.00 |
| HD1840-R5 | HD1840 | 5 | 23 | 2.95 | 1.28 | 0.94 | 4.64 | 8.59 | 15.29 |
| HD1840-R6 | HD1840 | 6 | 23 | 3.02 | 1.31 | 0.96 | 4.74 | 8.89 | 15.77 |
| HD1841-R1 | HD1841 | 1 | 10 | 2.25 | 0.98 | 0.98 | 4.62 | 8.27 | 10.00 |
| HD1841-R3 | HD1841 | 3 | 6 | 1.63 | 0.71 | 0.91 | 3.70 | 5.49 | 6.00 |
| HD1841-R4 | HD1841 | 4 | 16 | 2.60 | 1.13 | 0.94 | 4.50 | 8.00 | 13.20 |
| HD1841-R5 | HD1841 | 5 | 30 | 3.22 | 1.40 | 0.95 | 4.71 | 8.85 | 16.04 |
| HD1841-R6 | HD1841 | 6 | 25 | 2.99 | 1.30 | 0.93 | 4.61 | 8.42 | 14.54 |
| HD1842-R1 | HD1842 | 1 | 49 | 3.05 | 1.32 | 0.78 | 4.29 | 7.40 | 12.03 |
| HD1842-R2 | HD1842 | 2 | 7 | 1.75 | 0.76 | 0.90 | 3.98 | 7.00 | 7.00 |
| HD1842-R3 | HD1842 | 3 | 14 | 2.60 | 1.13 | 0.98 | 4.83 | 9.25 | 14.00 |
| HD1842-R4 | HD1842 | 4 | 8 | 1.91 | 0.83 | 0.92 | 4.11 | 6.99 | 8.00 |
| HD1842-R5 | HD1842 | 5 | 12 | 2.40 | 1.04 | 0.96 | 4.64 | 8.55 | 12.00 |
| HD1843-R1 | HD1843 | 1 | 40 | 3.36 | 1.46 | 0.91 | 4.65 | 8.54 | 14.76 |
| HD1843-R3 | HD1843 | 3 | 44 | 2.90 | 1.26 | 0.77 | 4.02 | 6.94 | 11.85 |
| HD1843-R4 | HD1843 | 4 | 22 | 2.90 | 1.26 | 0.94 | 4.61 | 8.40 | 14.43 |
| HD1843-R5 | HD1843 | 5 | 16 | 2.61 | 1.13 | 0.94 | 4.46 | 7.77 | 12.12 |
| HD1843-R6 | HD1843 | 6 | 21 | 2.89 | 1.26 | 0.95 | 4.65 | 8.51 | 14.42 |
| HD1844-R2 | HD1844 | 2 | 17 | 2.67 | 1.16 | 0.94 | 4.51 | 8.05 | 13.30 |
| HD1844-R3 | HD1844 | 3 | 27 | 3.10 | 1.35 | 0.94 | 4.66 | 8.59 | 14.81 |
| HD1844-R4 | HD1844 | 4 | 22 | 2.91 | 1.26 | 0.94 | 4.63 | 8.51 | 14.84 |
| HD1844-R6 | HD1844 | 6 | 22 | 2.83 | 1.23 | 0.92 | 4.53 | 8.11 | 13.69 |
| HD1844-R7 | HD1844 | 7 | 21 | 2.95 | 1.28 | 0.97 | 4.77 | 9.00 | 16.17 |
| HD1845-R1 | HD1845 | 1 | 7 | 1.76 | 0.76 | 0.90 | 3.74 | 5.54 | 7.00 |
| HD1845-R2 | HD1845 | 2 | 11 | 2.25 | 0.98 | 0.94 | 4.40 | 7.74 | 11.00 |
| HD1845-R3 | HD1845 | 3 | 8 | 2.04 | 0.89 | 0.98 | 4.72 | 8.00 | 8.00 |
| HD1845-R5 | HD1845 | 5 | 18 | 2.67 | 1.16 | 0.92 | 4.50 | 8.14 | 14.24 |
| HD1845-R6 | HD1845 | 6 | 29 | 3.27 | 1.42 | 0.97 | 4.80 | 9.12 | 16.45 |
| HD1846-R1 | HD1846 | 1 | 7 | 1.73 | 0.75 | 0.89 | 3.74 | 5.78 | 7.00 |
| HD1846-R2 | HD1846 | 2 | 19 | 2.39 | 1.04 | 0.81 | 3.96 | 6.62 | 11.10 |
| HD1846-R3 | HD1846 | 3 | 10 | 1.35 | 0.59 | 0.59 | 2.63 | 4.16 | 6.95 |
| HD1846-R4 | HD1846 | 4 | 8 | 1.91 | 0.83 | 0.92 | 4.11 | 6.99 | 8.00 |
| HD1846-R6 | HD1846 | 6 | 6 | 1.75 | 0.76 | 0.98 | 4.52 | 6.00 | 6.00 |
| HD1847-R2 | HD1847 | 2 | 21 | 2.66 | 1.15 | 0.87 | 4.25 | 7.47 | 12.81 |
| HD1847-R3 | HD1847 | 3 | 15 | 2.37 | 1.03 | 0.87 | 4.14 | 6.92 | 10.88 |
| HD1847-R5 | *HD1847* | *5* | *26* | *2.97* | *1.29* | *0.91* | *4.54* | *8.14* | *13.48* |
| HD1847-R7 | HD1847 | 7 | 16 | 2.23 | 0.97 | 0.81 | 3.84 | 6.23 | 9.73 |
| SCB-006 | S0440 | 1 | 11 | 1.82 | 0.79 | 0.76 | 3.39 | 5.44 | 8.56 |
| SCB-007 | S0440 | 2 | 25 | 2.75 | 1.20 | 0.86 | 4.26 | 7.39 | 12.31 |
| SCB-010 | S0440 | 3 | 16 | 2.32 | 1.01 | 0.84 | 3.99 | 6.53 | 10.16 |
| SCB-011 | S0440 | 4 | 13 | 2.04 | 0.88 | 0.79 | 3.65 | 5.68 | 8.46 |
| SCB-015 | S0440 | 5 | 17 | 2.33 | 1.01 | 0.82 | 3.89 | 6.68 | 11.41 |
| SCB-016 | S0440 | 6 | 12 | 2.20 | 0.95 | 0.88 | 4.07 | 6.67 | 10.28 |
| SCB-019 | S0440 | 7 | 24 | 2.86 | 1.24 | 0.90 | 4.45 | 7.87 | 13.01 |
| SCB-026 | S0440 | 8 | 24 | 2.85 | 1.24 | 0.90 | 4.42 | 7.90 | 13.58 |
| SCB-075 | S0443 | 1 | 5 | 1.56 | 0.68 | 0.97 | 4.33 | 5.00 | 5.00 |
| SCB-076 | S0443 | 2 | 4 | 0.51 | 0.22 | 0.37 | 1.63 | 2.25 | 3.50 |
| SCB-077 | S0443 | 3 | 6 | 1.67 | 0.72 | 0.93 | 4.11 | 6.00 | 6.00 |
| SCB-085 | S0443 | 4 | 11 | 2.00 | 0.87 | 0.83 | 3.75 | 6.15 | 9.70 |
| SCB-087 | S0443 | 5 | 14 | 2.38 | 1.04 | 0.90 | 4.28 | 7.39 | 12.29 |
| SCB-090 | S0443 | 6 | 6 | 1.19 | 0.52 | 0.66 | 2.64 | 4.03 | 6.00 |
| SCB-095 | S0443 | 7 | 14 | 1.78 | 0.77 | 0.68 | 3.23 | 4.68 | 6.73 |
| SCB-096 | S0443 | 8 | 16 | 2.33 | 1.01 | 0.84 | 4.02 | 6.72 | 10.90 |
| SCB-099 | S0444 | 1 | 3 | 1.10 | 0.48 | 1.00 | 3.00 | 3.00 | 3.00 |
| SCB-107 | S0444 | 3 | 2 | 0.69 | 0.30 | 1.00 | 2.00 | 2.00 | 2.00 |
| SCB-116 | S0444 | 4 | 2 | 0.64 | 0.28 | 0.92 | 2.00 | 2.00 | 2.00 |
| SCB-117 | S0444 | 5 | 4 | 1.24 | 0.54 | 0.90 | 3.50 | 4.00 | 4.00 |
| SCB-119 | S0444 | 6 | 10 | 2.11 | 0.92 | 0.92 | 4.16 | 6.80 | 10.00 |
| SCB-127 | S0444 | 8 | 5 | 1.30 | 0.57 | 0.81 | 3.22 | 5.00 | 5.00 |
| SCB-134 | S0445 | 1 | 9 | 2.20 | 0.95 | 1.00 | 5.00 | 9.00 | 9.00 |
| SCB-135 | S0445 | 2 | 4 | 1.39 | 0.60 | 1.00 | 4.00 | 4.00 | 4.00 |
| SCB-145 | S0445 | 3 | 4 | 1.39 | 0.60 | 1.00 | 4.00 | 4.00 | 4.00 |
| SCB-146 | S0445 | 4 | 6 | 1.70 | 0.74 | 0.95 | 3.97 | 6.00 | 6.00 |
| SCB-151 | S0445 | 5 | 5 | 1.61 | 0.70 | 1.00 | 5.00 | 5.00 | 5.00 |
| SCB-152 | S0445 | 6 | 17 | 2.75 | 1.20 | 0.97 | 4.75 | 8.91 | 15.81 |
| SCB-154 | S0445 | 7 | 5 | 1.61 | 0.70 | 1.00 | 5.00 | 5.00 | 5.00 |
| SCB-155 | S0445 | 8 | 3 | 1.05 | 0.46 | 0.96 | 3.00 | 3.00 | 3.00 |
| SCB-208 | S0448 | 1 | 27 | 1.70 | 0.74 | 0.52 | 2.81 | 4.29 | 6.63 |
| SCB-218 | S0448 | 3 | 15 | 2.54 | 1.10 | 0.94 | 4.46 | 7.86 | 12.77 |
| SCB-223 | S0448 | 4 | 14 | 2.36 | 1.03 | 0.89 | 4.18 | 6.98 | 10.84 |
| SCB-224 | S0448 | 5 | 17 | 2.50 | 1.09 | 0.88 | 4.23 | 7.30 | 12.06 |
| SCB-227 | S0448 | 6 | 12 | 2.19 | 0.95 | 0.88 | 4.07 | 6.66 | 10.37 |
| SCB-229 | S0448 | 7 | 8 | 1.97 | 0.86 | 0.95 | 4.33 | 7.46 | 8.00 |
| SCB-329 | S0452 | 1 | 20 | 2.60 | 1.13 | 0.87 | 4.24 | 7.19 | 11.31 |
| SCB-330 | S0452 | 2 | 24 | 2.82 | 1.23 | 0.89 | 4.41 | 7.75 | 12.82 |
| SCB-331 | S0452 | 3 | 24 | 2.60 | 1.13 | 0.82 | 4.07 | 6.88 | 11.09 |
| SCB-332 | S0452 | 4 | 16 | 2.29 | 0.99 | 0.83 | 3.96 | 6.32 | 9.37 |
| SCB-333 | S0452 | 5 | 21 | 2.80 | 1.22 | 0.92 | 4.48 | 7.97 | 13.17 |
| SCB-342 | S0452 | 6 | 23 | 2.63 | 1.14 | 0.84 | 4.16 | 7.00 | 11.13 |
| SCB-343 | S0452 | 7 | 16 | 2.13 | 0.92 | 0.77 | 3.66 | 5.88 | 9.23 |

Table A3. Z-statistic and associated p-values for pairwise comparisons (Benjamini-Hochberg) between macrofaunal density of substrates –FeMn crust, phosphorite, basalt, and sedimentary rocks. Asterisks (*) indicate statistical significance at a 2.5% level.

| Substrate type | Basalt | FeMn crust | Phosphorite |
| --- | --- | --- | --- |
| FeMn crust | Z: 4.48 |  |  |
|  | p: 0.0001* |  |  |
| Phosphorite | Z: -0.013 | Z: -4.49 |  |
|  | p: 0.49 | p: 0.0001* |  |
| Sedimentary | Z: 1.32 | Z: -1.64 | Z: 1.33 |
|  | p: 0.11 | p: 0.09 | p: 0.13 |

Table A4. Z-statistic and associated p-values for pairwise comparisons (Benjamini-Hochberg) between Shannon-Weiner diversity (H’_[loge]_ and H’_[log10]_) of substrates –FeMn crust, phosphorite, basalt, and sedimentary rocks. Asterisks (*) indicate statistical significance at a 2.5% level.

| Substrate type | Basalt | FeMn crust | Phosphorite |
| --- | --- | --- | --- |
| FeMn crust | Z: 2.82 |  |  |
|  | p: 0.0047* |  |  |
| Phosphorite | Z: -2.16 | Z: -5.31 |  |
|  | p: 0.0227* | p: 0.0000* |  |
| Sedimentary | Z: -1.04 | Z: -3.06 | Z: 0.54 |
|  | p: 0.17 | p: 0.0033* | p: 0.29 |

Table A5. T-statistic and p-value of pairwise test from PERMANOVA for macrofaunal community composition between substrate types (df=3).

| Substrate type | FeMn crust | Basalt | Sedimentary |
| --- | --- | --- | --- |
| Basalt | t: 0.89,  p: 0.82 | - | - |
| Sedimentary | t: 1.19,  p: 0.043 | t: 1.19,  p: 0.111 | - |
| Phosphorite | t: 1.05,  p: 0.27 | t: 1.61,  p: 0.001 | t: 1.53,  p: 0.001 |

Table A6. List of all 200 species on FeMn crusts and their frequency of occurrence (number of rocks they were found on).

| Phylum | Species morphotype | Frequency of occurrence |
| --- | --- | --- |
| Echinodermata | *Ophiocten* cf. *centobi* | 12 |
| Arthropoda | *Pseudotanais* sp. 1 | 9 |
| Echinodermata | *Ophioleuce* cf. *gracilis* | 9 |
| Echinodermata | Ophiuroidea sp. 5 (postlarvae) | 8 |
| Annelida | Spirorbinae sp. 1 | 7 |
| Annelida | *Chloeia* spp. | 6 |
| Arthropoda | *Munnopsurus* sp. 1 | 6 |
| Echinodermata | Ophiuroidea sp. 7 (postlarvae) | 6 |
| Porifera | Porifera sp. 5 | 6 |
| Annelida | Serpulidae spp. (juvenile) | 5 |
| Arthropoda | Munnidae sp. 1 | 5 |
| Bryozoa | Bryozoa sp. 5 | 5 |
| Cnidaria | Actiniaria | 5 |
| Echinodermata | *Astrophiura marionae* | 5 |
| Echinodermata | Ophiuroidea sp. 13 | 5 |
| Echinodermata | Ophiuroidea sp. 6 (postlarvae) | 5 |
| Mollusca | Tellinidae sp. 1 | 5 |
| Annelida | Maldanidae sp. 1 | 4 |
| Annelida | Flabelligeridae sp. 1 | 4 |
| Arthropoda | Acari sp. 4 | 4 |
| Arthropoda | Stenothoidae sp. 3 | 4 |
| Arthropoda | Paramunnidae sp. 1 | 4 |
| Bryozoa | Bryozoa sp. 8 | 4 |
| Echinodermata | Ophiuroidea sp. 11 | 4 |
| Mollusca | Gastropoda sp. 11 | 4 |
| Porifera | Porifera sp. 15 | 4 |
| Porifera | Porifera sp. 29 | 4 |
| Annelida | Syllidae sp. 6 (juvenile) | 3 |
| Annelida | Sabellidae sp. 1 | 3 |
| Annelida | *Spio*? Spp. | 3 |
| Annelida | *Aphelochaeta* spp. (juvenile) | 3 |
| Arthropoda | Acari sp. 1 | 3 |
| Arthropoda | Acari sp. 3 | 3 |
| Arthropoda | *Paraleptognathia bisetulosa* | 3 |
| Echinodermata | Ophiuroidea sp. 1 (juvenile) | 3 |
| Mollusca | Aplacophora sp. 2 | 3 |
| Mollusca | Gastropoda sp. 22 | 3 |
| Mollusca | Monoplacophora sp. 1 | 3 |
| Porifera | Porifera sp. 20 | 3 |
| Porifera | Porifera sp. 30 | 3 |
| Annelida | *Ophryotrocha* spp. | 2 |
| Annelida | *Lepidonotus* sp. 1 | 2 |
| Annelida | Polynoidae sp. 4 | 2 |
| Annelida | Polynoidae sp. 5 (juvenile) | 2 |
| Annelida | *Eusyllis* sp. 1 | 2 |
| Annelida | *Salmacina tribranchiata* sp. 1 | 2 |
| Annelida | *Laonice* sp. 1 (juvenile) | 2 |
| Arthropoda | Oedicerotidae sp. 1 | 2 |
| Arthropoda | *Gnathia* sp. 1 | 2 |
| Arthropoda | Munnopsidae sp. 1 | 2 |
| Arthropoda | Nannoniscidae sp. 1 | 2 |
| Arthropoda | Tridentellidae sp. 1 (juvenile) | 2 |
| Arthropoda | Unid. Isopoda sp. 1 | 2 |
| Arthropoda | *Araphura* sp.1 | 2 |
| Bryozoa | Bryozoa sp. 13 | 2 |
| Mollusca | Aplacophora sp. 1 | 2 |
| Mollusca | Aplacophora sp. 3 | 2 |
| Mollusca | Bivalvia sp. 4 (juvenile) | 2 |
| Mollusca | Gastropoda sp. 3 | 2 |
| Mollusca | Limpet sp. 1 | 2 |
| Mollusca | Scaphopoda sp. 1 | 2 |
| Porifera | Demospongiae sp. 1 | 2 |
| Porifera | Demospongiae sp. 2 | 2 |
| Porifera | Porifera sp. 10 | 2 |
| Porifera | Porifera sp. 27 | 2 |
| Annelida | *Notomastus* sp. 1 | 1 |
| Annelida | *Clymenella californica* | 1 |
| Annelida | Maldanidae sp. 3 | 1 |
| Annelida | *Lumbrineris* nr. *inflata* | 1 |
| Annelida | *Lumbrineris* nr. *latreilli* | 1 |
| Annelida | Lumbrineridae sp. 2 | 1 |
| Annelida | Chrysopetalidae? sp. 1 | 1 |
| Annelida | *Gyptis* sp. 1 | 1 |
| Annelida | *Podarkeopsis perkinsi* | 1 |
| Annelida | Hesionidae sp. 1 (juvenile) | 1 |
| Annelida | Nereididae sp. | 1 |
| Annelida | *Eulalia (Sige)* nr*. bifoliata* | 1 |
| Annelida | Phyllodocidae (fragment) | 1 |
| Annelida | Phyllodocidae sp. 1 | 1 |
| Annelida | *Harmothoe* nr. *imbricata* | 1 |
| Annelida | *Lepidasthenia* sp. 1 | 1 |
| Annelida | *Malmgreniella* nr. *baschi* | 1 |
| Annelida | Polynoidae sp. 1 | 1 |
| Annelida | Polynoidae sp. 3 | 1 |
| Annelida | Sphaerodoridae sp. 3 | 1 |
| Annelida | Sphaerodoridae sp. | 1 |
| Annelida | *Dioplosyllis* nr. *Tridentata* | 1 |
| Annelida | *Dioplosyllis* sp. 1 | 1 |
| Annelida | *Dioplosyllis* sp. 3 | 1 |
| Annelida | Eusyllinae sp. 1 | 1 |
| Annelida | *Exogone (parexogone)*? sp. 1 | 1 |
| Annelida | *Sphaerosyllis* nr. *ranunculus* | 1 |
| Annelida | Exogoninae sp. 1 | 1 |
| Annelida | Syllidae sp. 2 (juvenile) | 1 |
| Annelida | Syllidae sp. 3 | 1 |
| Annelida | *Protis*? sp. 1 | 1 |
| Annelida | Serpulidae sp. 3 | 1 |
| Annelida | Serpulidae sp. 4 | 1 |
| Annelida | Serpulidae sp. 5 | 1 |
| Annelida | Serpulidae sp. 6 (juvenile) | 1 |
| Annelida | Polydoridae sp. 1 | 1 |
| Annelida | *Laonice* nr. *nuchula* | 1 |
| Annelida | *Paraprionospio*? (juvenile) | 1 |
| Annelida | Spionidae (fragments) | 1 |
| Annelida | Ampharetidae sp. 1 (juvenile) | 1 |
| Annelida | *Chaetozone* sp. 1 | 1 |
| Annelida | Cirratulidae sp. 3 | 1 |
| Annelida | Cirratulidae? (fragments) | 1 |
| Annelida | *Pherusa* sp. 1 | 1 |
| Annelida | *Trophoniella*? sp. 1 | 1 |
| Annelida | Flabelligeridae fragment | 1 |
| Annelida | *Pista* nr. *Elongata* | 1 |
| Annelida | Terebellidae sp. 4 | 1 |
| Annelida | Terebellidae spp. (juvenile) | 1 |
| Annelida | Trichobranchidae sp. 1 (juvenile) | 1 |
| Annelida | Polychaeta fragment | 1 |
| Arthropoda | Acari sp. 2 | 1 |
| Arthropoda | Acari sp. 5 | 1 |
| Arthropoda | Caprellidae sp. 1 | 1 |
| Arthropoda | Dulichiidae sp. 1 | 1 |
| Arthropoda | *Rhachotropis* *inflata* | 1 |
| Arthropoda | Paradaliscidae sp. 1 | 1 |
| Arthropoda | *Rhynohalicella* *hadona* | 1 |
| Arthropoda | Photidae sp. 1 | 1 |
| Arthropoda | Stegocephalidae sp. 2 | 1 |
| Arthropoda | Stegocephalidaes p. 4 (juvenile) | 1 |
| Arthropoda | *Metopa* nr. *dawsoni* | 1 |
| Arthropoda | *Stenothoe* *frecanda* | 1 |
| Arthropoda | Galatheidae sp. 1 | 1 |
| Arthropoda | Munididae sp. 1 | 1 |
| Arthropoda | Munnidae sp. 3 | 1 |
| Arthropoda | Penaeidae sp. 1 | 1 |
| Arthropoda | Unid. shrimp larvae sp. 1 | 1 |
| Arthropoda | Cirolanidae sp. 1 | 1 |
| Arthropoda | *Desmosoma* sp. 1 | 1 |
| Arthropoda | Haploniscidae sp. 1 | 1 |
| Arthropoda | Haploniscidae sp. 2 | 1 |
| Arthropoda | *Pleurogonium* sp. 1 | 1 |
| Arthropoda | *Tridentella* *glutocantha* | 1 |
| Arthropoda | Unid. Isopoda sp. 2 (juvenile) | 1 |
| Arthropoda | Anarthruridae sp. 1 | 1 |
| Arthropoda | *Araphura* sp. 2 | 1 |
| Arthropoda | Cirripedia sp. 1 | 1 |
| Bryozoa | Bryozoa sp. 6 | 1 |
| Bryozoa | Bryozoa sp. 9 | 1 |
| Chordata | *Megalodicopia* sp. | 1 |
| Cnidaria | Actiniaria? | 1 |
| Cnidaria | Dendrophylliidae sp. 1 | 1 |
| Echinodermata | *Ypsilothuria* sp. 1 | 1 |
| Echinodermata | *Amphipholis* *pugetana*? (juvenile) | 1 |
| Echinodermata | *Ophiacantha (Ophiotreta)* sp? | 1 |
| Echinodermata | *Ophiacantha normani* | 1 |
| Echinodermata | Ophiuroidea sp. 12 | 1 |
| Echinodermata | Ophiuroidea sp. 14 | 1 |
| Echinodermata | Ophiuroidea sp. 15 | 1 |
| Echinodermata | Ophiuroidea sp. 3 (juvenile) | 1 |
| Echinodermata | Ophiuroidea sp. 4 (juvenile) | 1 |
| Echinodermata | Ophiuroidea sp. 8 (postlarvae) | 1 |
| Echinodermata | Unidentified Ophiuroidea | 1 |
| Mollusca | Aplacophora sp. 4 | 1 |
| Mollusca | Thyasiridae sp. 2 | 1 |
| Mollusca | *Placopecten* sp. 1 | 1 |
| Mollusca | Bivalvia sp. 1 | 1 |
| Mollusca | *Scutopus* sp. 1 | 1 |
| Mollusca | Neolepetopsidae sp. 1 | 1 |
| Mollusca | Gastropoda sp. 1 | 1 |
| Mollusca | Gastropoda sp. 12 | 1 |
| Mollusca | Gastropoda sp. 14 | 1 |
| Mollusca | Gastropoda sp. 18 | 1 |
| Mollusca | Gastropoda sp. 19 | 1 |
| Mollusca | Gastropoda sp. 2 | 1 |
| Mollusca | Gastropoda sp. 4 | 1 |
| Mollusca | Gastropoda sp. 5 | 1 |
| Mollusca | Gastropoda sp. 6 | 1 |
| Mollusca | Gastropoda sp. 7 | 1 |
| Mollusca | Gastropoda sp. 8 | 1 |
| Mollusca | Limpet sp. 6 | 1 |
| Mollusca | Limpet sp. 7 | 1 |
| Mollusca | Limpet sp. 9 (juvenile) | 1 |
| Mollusca | Polyplacophora sp. 3 | 1 |
| Mollusca | *Spiomenia spiculata* | 1 |
| Mollusca | *Macranelle* sp. 1 | 1 |
| Nemertea | Planaria | 1 |
| Porifera | *Asbestopluma* sp. 1 | 1 |
| Porifera | *Hexactinella* sp. 2 | 1 |
| Porifera | *Hexactinella* sp. 3 | 1 |
| Porifera | *Hexactinella* sp. 6 | 1 |
| Porifera | *Hexactinella* sp. 7 | 1 |
| Porifera | *Hexactinella* sp. 8 | 1 |
| Porifera | Porifera sp. 1 | 1 |
| Porifera | Porifera sp. 11 | 1 |
| Porifera | Porifera sp. 12 | 1 |
| Porifera | Porifera sp. 17 | 1 |
| Porifera | Porifera sp. 23 | 1 |
| Porifera | Porifera sp. 25 | 1 |
| Porifera | Porifera sp. 26 | 1 |
| Porifera | Porifera sp. 28 | 1 |
| Porifera | Porifera sp. 3 | 1 |
| Porifera | Porifera sp. 4 | 1 |
| Porifera | Porifera sp. 7 | 1 |

Table A7. Species unique to FeMn crusts and total number of individuals collected in this study.

| Phylum | Species morphotype | Number of individuals |
| --- | --- | --- |
| Annelida | *Clymenella californica* | 2 |
| Annelida | Maldanidae sp. 3 | 1 |
| Annelida | Lumbrineridae sp. 2 | 1 |
| Annelida | Chrysopetalidae? sp. 1 | 1 |
| Annelida | Phyllodocidae (fragment) | 1 |
| Annelida | Phyllodocidae sp. 1 | 1 |
| Annelida | Polynoidae sp. 3 | 1 |
| Annelida | Sphaerodoridae sp. 3 | 1 |
| Annelida | *Dioplosyllis* sp. 3 | 2 |
| Annelida | *Eusyllinae* sp. 1 | 1 |
| Annelida | *Exogone* "*parexogone*"? sp. 1 | 1 |
| Annelida | Exogoninae sp. 1 | 3 |
| Annelida | Syllidae sp. 2 (juvenile) | 1 |
| Annelida | Syllidae sp. 3 | 2 |
| Annelida | Syllidae sp. 6 (juvenile) | 3 |
| Annelida | *Protis*? sp. 1 | 1 |
| Annelida | *Salmacina tribranchiata* sp. 1 | 3 |
| Annelida | Serpulidae sp. 3 | 1 |
| Annelida | Serpulidae sp. 5 | 2 |
| Annelida | Serpulidae spp. (juvenile) | 28 |
| Annelida | *Laonice* nr. *nuchula* | 1 |
| Annelida | *Paraprionospio*? (juvenile) | 1 |
| Annelida | Spionidae (fragments) | 1 |
| Annelida | Ampharetidae sp. 1 (juvenile) | 3 |
| Annelida | *Aphelochaeta* spp. (juvenile) | 3 |
| Annelida | *Chaetozone* sp. 1 | 1 |
| Annelida | *Trophoniella*? sp. 1 | 1 |
| Annelida | Flabelligeridae fragment | 1 |
| Annelida | *Pista* nr. *elongata* | 2 |
| Annelida | Terebellidae spp. (juvenile) | 1 |
| Annelida | Polychaeta fragment | 2 |
| Arthropoda | Acari sp. 5 | 1 |
| Arthropoda | Paradaliscidae sp. 1 | 1 |
| Arthropoda | *Rhynohalicella hadona* | 2 |
| Arthropoda | Photidae sp. 1 | 29 |
| Arthropoda | Stegocephalidae sp. 2 | 1 |
| Arthropoda | Penaeidae sp. 1 | 1 |
| Arthropoda | Cirolanidae sp. 1 | 1 |
| Arthropoda | *Desmosoma* sp. 1 | 1 |
| Arthropoda | Haploniscidae sp. 1 | 2 |
| Arthropoda | Haploniscidae sp. 2 | 1 |
| Arthropoda | Nannoniscidae sp. 1 | 2 |
| Arthropoda | Paramunnidae sp. 1 | 5 |
| Arthropoda | *Tridentella glutocantha* | 2 |
| Arthropoda | Unid. Isopoda sp. 1 | 2 |
| Arthropoda | *Paraleptognathia bisetulosa* | 7 |
| Arthropoda | Anarthruridae sp. 1 | 3 |
| Arthropoda | *Araphura* sp. 2 | 3 |
| Bryozoa | Bryozoa sp.6 | 4 |
| Chordata | *Megalodicopia* sp. | 1 |
| Cnidaria | Actiniaria? | 1 |
| Cnidaria | Dendrophylliidae sp. 1 | 2 |
| Echinodermata | *Ypsilothuria* sp. 1 | 1 |
| Echinodermata | *Ophiacantha normani* | 1 |
| Echinodermata | Ophiuroidea sp. 1 (juvenile) | 3 |
| Echinodermata | Ophiuroidea sp. 14 | 1 |
| Echinodermata | Ophiuroidea sp. 15 | 1 |
| Echinodermata | Ophiuroidea sp. 3 (juvenile) | 1 |
| Echinodermata | Ophiuroidea sp. 4 (juvenile) | 1 |
| Echinodermata | Ophiuroidea sp. 8 (postlarvae) | 2 |
| Echinodermata | Unidentified Ophiuroidea | 1 |
| Mollusca | Aplacophora sp. 4 | 3 |
| Mollusca | Tellinidae sp. 1 | 6 |
| Mollusca | Thyasiridae sp. 2 | 1 |
| Mollusca | Bivalvia sp. 1 | 1 |
| Mollusca | *Scutopus* sp. 1 | 1 |
| Mollusca | Neolepetopsidae sp. 1 | 1 |
| Mollusca | Gastropoda sp. 1 | 1 |
| Mollusca | Gastropoda sp. 12 | 1 |
| Mollusca | Gastropoda sp. 18 | 1 |
| Mollusca | Gastropoda sp. 19 | 2 |
| Mollusca | Gastropoda sp. 2 | 1 |
| Mollusca | Gastropoda sp. 22 | 3 |
| Mollusca | Gastropoda sp. 3 | 2 |
| Mollusca | Gastropoda sp. 4 | 1 |
| Mollusca | Gastropoda sp. 6 | 1 |
| Mollusca | Gastropoda sp. 7 | 1 |
| Mollusca | Gastropoda sp. 8 | 1 |
| Mollusca | Limpet sp. 1 | 4 |
| Mollusca | Limpet sp. 7 | 1 |
| Mollusca | Limpet sp. 9 (juvenile) | 1 |
| Mollusca | Scaphopoda sp. 1 | 2 |
| Mollusca | *Spiomenia spiculata* | 2 |
| Porifera | *Hexactinella* sp. 2 | 1 |
| Porifera | *Hexactinella* sp. 3 | 1 |
| Porifera | *Hexactinella* sp. 6 | 1 |
| Porifera | Porifera sp. 11 | 1 |
| Porifera | Porifera sp. 12 | 1 |
| Porifera | Porifera sp. 17 | 1 |
| Porifera | Porifera sp. 20 | 3 |
| Porifera | Porifera sp. 23 | 1 |
| Porifera | Porifera sp. 25 | 1 |
| Porifera | Porifera sp. 26 | 1 |
| Porifera | Porifera sp. 27 | 2 |
| Porifera | Porifera sp. 28 | 1 |

Table A8. Species unique to phosphorite rocks and total number of individuals collected in this study.

| Phylum | Species morphotype | Number of individuals | |
| --- | --- | --- | --- |
| Annelida | Amphinomidae sp. 1 | | 3 |
| Annelida | Notomastus spp. (juvenile) | | 1 |
| Annelida | Maldanidae sp. 4 | | 1 |
| Annelida | Maldanidae sp. 5 | | 2 |
| Annelida | Paraonella? Sp. 1 (juvenile) | | 2 |
| Annelida | Paraonidae sp. 1 | | 5 |
| Annelida | *Schistomeringos* sp. 1 | | 2 |
| Annelida | *Eunice* sp. 1 | | 1 |
| Annelida | *Lumbrineris californiensis* | | 1 |
| Annelida | *Lumbrineris* nr. *japonica* | | 4 |
| Annelida | Myzostomidae sp. 1 | | 3 |
| Annelida | *Scalibregma californicum* | | 1 |
| Annelida | *Glycera nana* | | 1 |
| Annelida | *Gyptis* spp. | | 1 |
| Annelida | Nautiliniellidae? sp. 1 | | 1 |
| Annelida | *Nephtys* sp. 1 | | 6 |
| Annelida | *Phyllodoce* nr. *papillata* | | 1 |
| Annelida | *Harmothoe fragilis* | | 8 |
| Annelida | Polynoidae sp. 2 | | 3 |
| Annelida | *Eusyllis* nr. *blomstrandi* | | 2 |
| Annelida | *Pionosyllis*? sp. 1 | | 2 |
| Annelida | Syllidae sp. 5 | | 4 |
| Annelida | Syllidae? sp. 4 | | 1 |
| Annelida | *Pseudopotamilla* sp. 1 | | 3 |
| Annelida | Sabellidae sp. 2 | | 1 |
| Annelida | Serpulidae sp. 1 | | 2 |
| Annelida | Serpulidae sp. 7 | | 1 |
| Annelida | *Pseudopolydora* sp. 1 (juvenile) | | 4 |
| Annelida | *Prionospio ehlersi* | | 5 |
| Annelida | *Spiophanes berkeleyorum* | | 1 |
| Annelida | *Spiophanes*? sp. 2 (juvenile) | | 1 |
| Annelida | Spionidae larvae | | 1 |
| Annelida | *Cirriformia spirabrancha* | | 2 |
| Annelida | *Pherusa neopapillata* | | 1 |
| Annelida | *Pista* nr. *wui* | | 1 |
| Annelida | *Scionella* nr. *japonica* | | 1 |
| Annelida | Terebellidae sp. 1 (Neoamphitrite?)(Thelipus) | | 6 |
| Annelida | Terebellides sp. 1 | | 1 |
| Annelida | *Octobranchus* sp. 1 | | 1 |
| Arthropoda | Leuconidae sp. 1 | | 5 |
| Arthropoda | Caprellidae sp. 2 | | 5 |
| Arthropoda | Caprellidae sp. 4 | | 17 |
| Arthropoda | Pleustidae sp. 1 | | 6 |
| Arthropoda | Unid. Gammaridea | | 6 |
| Arthropoda | Majidae sp. 1 | | 1 |
| Arthropoda | *Gnathia tridens* | | 2 |
| Arthropoda | Munnopsidae sp. 2 | | 3 |
| Arthropoda | “*Nannoniscus*” sp. 1 | | 2 |
| Arthropoda | Paramunnidae sp. 2 | | 2 |
| Arthropoda | Agathotanaidae sp. 1 (juvenile) | | 4 |
| Arthropoda | Unid. Crustacea | | 1 |
| Arthropoda | Pycnogonidae sp. 2 | | 1 |
| Bryozoa | Bryozoa sp. 10 | | 2 |
| Bryozoa | Bryozoa sp. 12 | | 2 |
| Bryozoa | Bryozoa sp. 7 | | 4 |
| Cnidaria | Stylasteridae | | 1 |
| Echinodermata | Asteroidea | | 1 |
| Echinodermata | *Ophiacantha diplasia*? | | 1 |
| Echinodermata | *Ophiopholis longispana* | | 3 |
| Echinodermata | *Ophiosphalma*? (postlarvae) | | 1 |
| Echinodermata | Ophiuroidea sp. 16 | | 4 |
| Hemichordata | Hemichordata | | 1 |
| Mollusca | Gastropoda sp. 16 | | 1 |
| Mollusca | Gastropoda sp. 20 | | 1 |
| Mollusca | Limpet sp. 2 | | 3 |
| Mollusca | Limpet sp. 3 | | 1 |
| Mollusca | Polyplacophora sp. 2 | | 3 |
| Mollusca | Scaphopoda sp. 2 | | 2 |
| Porifera | Hexactinella sp. 4 | | 1 |
| Porifera | Porifera sp. 13 | | 1 |
| Porifera | Porifera sp. 21 | | 1 |
| Porifera | Porifera sp. 24 | | 1 |
