# Supplementary material for: Deep-ocean macrofaunal assemblages on ferromanganese and phosphorite-rich substrates in the Southern California Borderland
Source: PeerJ. 2024 Oct 31;12:e18290. doi: 10.7717/peerj.18290 (PMC11531752; doi:10.7717/peerj.18290)
Supplement: Supplemental Information 2 [file peerj-12-18290-s002.pdf]

Ryan A. McCarthy played a pivotal role in the data analysis and visualization for this manuscript. He developed key R scripts (mainly code) that were used to generate several figures central to the paper's results. Additionally, Ryan formatted all tables using LaTeX in Overleaf and handled other technical formatting tasks, such as correcting the references section after the first submission. His contributions were critical in ensuring that the figures and tables met publication standards. Initially, Ryan was at sea on a research cruise in Vietnam without email access, which made it impractical to involve him in the early submission discussions. And, he would not have been able to respond to PeerJ's email confirming co-authorship, delaying the process at that stage. To avoid delaying the first submission, we decided to wait until his return to have a thorough conversation regarding his co-authorship, ensuring his formal consent without postponing the paper's submission. If you would like more proof of his involvement, we have a word document with comments and edits he provided on an earlier version of the manuscript and we can submit that at your request.

Olivia S. Pereira's contributions were equally essential. She was involved in the project as a PhD student and was instrumental in developing the experimental design alongside co-author Dr. Lisa Levin before I (Michelle Guraieb) arrived as a master's student. While her early contributions were not fully recognized when I took over the project, upon reviewing PeerJ's co-authorship criteria again and reassessing her role, we realized her involvement clearly made her a co-author. Olivia was a crucial contributor to the statistical design in the early stages of the project when I was starting to develop my master's thesis, the original work from which this manuscript was developed. During the manuscript preparation and initial submission, Olivia was also at sea in Alaska with limited internet access, which led to her being acknowledged instead of listed as a co-author. After the first revision, she reviewed the manuscript, provided substantive edits (as indicated in the manuscript through comments, as requested), and contributed to the response letter.

All authors agree that both Olivia and Ryan should be recognized as co-authors due to the substantial intellectual and technical contributions they made to this work. We recognize that adding them as co-authors after the first submission is rare and we apologize for any confusion or misunderstandings this may have caused and sincerely hope that the journal will accept their co-authorship in recognition of their contributions.

Sincerely,

Michelle Guraieb

(On behalf of all authors)
